# John Tatham Thompson

**Published:** 1911-06

**Authors:** 


### Obituary.

### JOHN TATHAM THOMPSON, M.B., C.M. Edin.

THE news of the death of Mr. Tatham Thompson, at the early age of 52, will be received with sincere regret by his professional brethren and many other friends in Bristol and its neighbourhood, as well as throughout South Wales.

He was born at York, and educated at Bootham School where his father was senior master. The earlier days of his scientific career were spent in the laboratory of his distinguished brother Sylvanus, at that time Lecturer on Physics in University College, Bristol. He also worked at Pharmacy under the late Mr. G F. Schacht. He soon, however, passed on to Edinburgh as a student of medicine, and he took his medical degrees there in 1885.

He was a talented draughtsman, and often made sketches in association with programmes for University functions, concert cards and menus. He did drawings for Professor Sims Woodhead in his book on *Practical Pathology*, and also illustrated Dr. George Berry's handbook on *Diseases of the Eye.*

His early interest in eye diseases, and his exceptional delicacy of manipulation attracted the attention of Argyll-Robertson and his other teachers, and led to his election as Ophthalmic Surgeon to the Westminster Dispensary in Edinburgh.

In 1887 he was selected to fill the newly-created post of Ophthalmic Surgeon to the Cardiff Infirmary, and by his energy and talent he soon made a large Eye Department at the Institution, and so gained the confidence of the medical men in South Wales that he soon established a large private practice, and became a leading scientific opinion in his speciality.

He was a devoted worker in the best interests of the Cardiff Infirmary for twenty years, and his advice and skill were most highly appreciated throughout the district by rich and poor alike, for he was an accurate diagnostician, and an operator of exceptional delicacy. He was elected in turn to all the leading medical positions, and he was generally popular as a genial and gifted helper at social gatherings. He was a distinguished Freemason, and held many important offices in various orders of the Craft. He also did good service for the Territorial Army, and recently received a decoration from the King.

He made several important contributions to ophthalmic literature and practice.

He possessed an exceptional experience of Miners' Nystagmus, and read an important paper on this subject in 1890 at the Ophthalmological Society, which was followed by a speech by the late Simeon Snell of Sheffield, another authority on this matter.

These two also were early advocates of the use of the electro-magnet for removing iron fragments from the eyeball.

For some years Tatham Thompson suffered from poor health, his work was often done under painful conditions, somewhat straining a temper which was naturally most genial and merry; but he went bravely on until the malignant disease from which he suffered made it impossible for him to continue work, and he died after months of suffering on April 28th.

His professional brethren loved him, and held him in the very highest esteem; and the wide respect that he enjoyed from the public at large was shown by the number of those who attended or lined the route of his funeral.

We desire to express our sincere sympathy with his widow, a sister of a well-known Bristol student (Arthur Hill Joseph), and with his four young children.

